# Mutant p53 and TP53 mutations in esophageal squamous cell carcinoma: consistency and diagnostic significance

**DOI:** 10.3389/fgene.2026.1680422

**Published:** 2026-02-27

**Authors:** Xueyu Zhuang, Na Lin, Yanjuan Xu

**Affiliations:** Department of Pathology, The Second Affiliated Hospital of Fujian Medical University, Quanzhou, Fujian, China

**Keywords:** consistency analysis, diagnostic value, esophageal squamous cell carcinoma, p53, TP53, whole exome sequencing

## Abstract

**Background and objective:**

TP53 mutation is an initiating event in tumorigenesis in many cancers. Mutant p53 expression is an important manifestation of TP53 mutations, however, this association has not yet been confirmed in esophageal squamous cell carcinoma (ESCC). This study comprised three components. The first was screening for TP53 mutations using whole-exome sequencing (WES) or whole-genome sequencing (WGS). The second was identifying mutant p53 expression by immunohistochemical (IHC) staining to explore the association between mutant p53 expression and TP53 mutations. The third was assessing the diagnostic value of mutant p53 expression in patients with ESCC.

**Methods:**

Eighteen fresh ESCC specimens were collected for WES. For cases without TP53 mutations detected by WES, WGS was performed to confirm the results and identify additional mutations. These samples underwent p53 IHC staining, and p53 expression was assessed independently by two senior pathologists. The Kappa coefficient was used to evaluate interobserver consistency. An additional 60 ESCC samples and corresponding adjacent tissues were collected for IHC staining. The chi-square test was used to assess the diagnostic value of p53 expression.

**Results:**

WES revealed TP53 mutations in 13/18 cases. WGS of the five WES-negative samples identified TP53 mutations in four of them. Overall, TP53 mutations were detected in 17/18 cases (94.44%). Mutant p53 expression was present in all ESCC cases, and the consistency rate between TP53 mutation and p53 protein expression was 94.44% (17/18). In the combined WES + WGS cohort, mutant p53 expression was detected in 18/18 (100.00%) patients with ESCC. In the IHC cohort, mutant p53 expression was detected in 60/60 (100.00%) patients with ESCC. In both cohorts, Type I mutant p53 expression was the most common subtype, followed by Type IV. Type IV mutant expression was not observed in the WES + WGS cohort. The Kappa value for the two pathologists to was 0.954 (0.911–1.000). The sensitivity and specificity of mutant p53 expression for diagnosing ESCC were 1.00 (0.95–1.00) and 1.00 (0.95–1.00), respectively.

**Conclusion:**

Mutant p53 expression can serve as an alternative marker for TP53 mutation screening used WES or WGS. Mutant p53 expression shows high sensitivity and specificity in distinguishing ESCC and can assist in differentiating benign from malignant esophageal lesions. Five mutant p53 expression subtypes were identified in this study; however, their clinical significance requires further investigation.

## 1 Introduction

Esophageal cancer (EPC) is one of the most common malignant tumors in China (Zhu et al., 2023). Esophageal squamous cell carcinoma (ESCC) is the predominant type of EPC in Asia (Xiao et al., 2023). Current research indicates that the development of EPC is multifactorial, involving multiple pathways and stages, including the inactivation of tumor suppressor genes, activation of oncogenes, and disruption of the balance between cell proliferation and apoptosis (Xiao et al., 2023; Chang et al., 2025). A key characteristic of malignant tumors is their ability to proliferate (He et al., 2023). Therefore, an imbalance between cell proliferation and apoptosis plays an important role in tumorigenesis (Zhou et al., 2017). Abundant gene mutations are another hallmark of tumor development (Liu et al., 2006). EPCs are malignant digestive tract tumors characterized by a high frequency of genetic mutations (Cui et al., 2020).

More than 50% of human cancers harbor TP53 mutations, indicating that TP53 mutations are highly correlated with tumor development (Nian et al., 2024). TP53 mutation is the most common molecular event and an initiating factor for the occurrence of ESCC (Asl et al., 2023). The protein encoded by TP53, p53, is one of the most extensively studied and potent human tumor suppressor proteins, making it a valuable molecular target for the diagnosis and treatment of ESCC (Shimada, 2018). p53 suppresses tumor development by inhibiting cell proliferation, inducing apoptosis, and promoting DNA damage repair triggered by various intra- and intercellular stimuli (Feng et al., 2023). It has also been shown to interact with key cancer-related pathways, such as NOTCH1 and PI3K/AKT (Kim et al., 2007; Yugawa et al., 2007; Chen et al., 2022a; Abraham and O'Neill, 2014). In addition, the loss of functional p53 often occurs during the critical transition from benign adenoma to invasive carcinoma, contributing to tumor invasiveness (Hollstein et al., 1990). p53 mutations may also influence the tumor microenvironment and tumor metastasis (Nian et al., 2024; Zhao et al., 2025).

Previous studies have shown that the mutant p53 protein expression is highly consistent with the TP53 mutation status in gynecological tumors and breast cancer, suggesting that immunohistochemical (IHC) staining can serve as an effective alternative method for detecting TP53 mutations. However, it remains unclear whether a similar consistency exists between TP53 gene mutations and p53 protein expression patterns in esophageal epithelial lesions. It is also uncertain whether different observers can obtain consistent results when evaluating p53 protein IHC expression patterns.

This study explored the following key scientific questions: First, we aimed to clarify the association between TP53 gene mutation status and mutant p53 protein expression in ESCC. Second, we investigated the auxiliary diagnostic significance of mutant p53 protein expression in ESCC and adjacent tissues. Addressing these questions will provide an important theoretical basis and clinical guidance for the experimental design of molecular diagnosis approaches for ESCC.

## 2 Materials and methods

### 2.1 Study protocol

**FIGURE 1:**
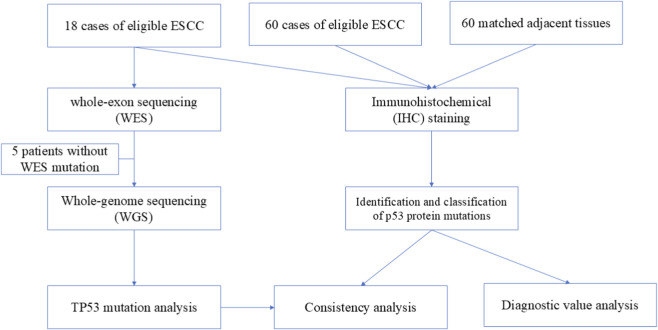
Flow chart of study design.

This study consisted of three parts. The first part involved screening for TP53 mutations using whole-exome sequencing (WES) or whole-genome sequencing (WGS) in 18 patients with ESCC (WES + WGS cohort). The second part aimed to identify mutant-expressed p53 protein through IHC staining to evaluate the association between mutant-expressed p53 and TP53 gene mutations in the same 18 patients with ESCC. Third, we assessed the diagnostic value of mutant p53 expression in 60 additional patients with ESCC (IHC cohort) using the combined total of 78 patients admitted to Fujian Medical University Cancer Hospital between January 2023 and July 2024. All paraffin-embedded tissues were obtained from surgical specimens donated by patients who provided written informed consent. This study was approved by the ethics committee of the Second Affiliated Hospital of Fujian Medical University (2022–237). A flowchart of the study design is shown in Figure 1.

Patients included in this study were required to (1) have pathologically confirmed ESCC and have undergone radical resection; (2) have any TNM stage according to the eighth edition of the American Joint Committee on Cancer; (3) be ≥18 years of age; and (4) have provided informed consent. Exclusion criteria were any preoperative treatment (including radiotherapy, chemotherapy, or immunotherapy) or the presence of double or multiple cancers.

### 2.2 The experimental process of WES

Genomic DNA was extracted from each sample, and a library was constructed following electrophoresis quality assessment. All qualified DNA samples were randomly sheared into 150–220 bp fragments using Covaris, after which library construction and target capture were performed with the Agilent SureSelect Human All Exon V8 kit. The DNA fragments underwent terminal repair, ploy (A) tail addition, sequencing adapter ligation, purification, magnetic bead capture, PCR amplification, and library preparation. After library quality inspection, paired-end sequencing was performed using a high-throughput sequencer.

Sequencing data were aligned to the Human Reference Genome (human_B37), and single nucleotide polymorphisms (SNP) were identified using *SAMtools*. Variant sites were annotated using Qualimap software. Insertions and deletions (InDels) were detected with *GATK4* software based on alignment to the reference genome. The SNP and InDel detection results were annotated using *Annovar* software.

### 2.3 The experimental process of WGS

All samples were collected by senior physicians to ensure that specimen condition and quality met the pre-sequencing requirements. WGS was conducted using the Ruijian detection kit, nucleic acid extraction reagent, and a universal sequencing reaction kit purchased from Hangzhou Ruipu Gene Company. Sequencing was carried out on the Illumina HiSeq 4000 platform. Tissue quality control criteria were as follows: malignant tumor cell proportion sr0%, total DNA ≥ s0 ng, average sequencing depth ≥ s000, and ≥80% of the genome achieving no less than 10% of the average sequencing depth.

### 2.4 IHC assay of p53

Four-micrometer–thick paraffin-embedded ESCC tissue sections were re-cut from samples stored in the pathology department and mounted on silane-coated slides. IHC staining was conducted by Na Lin. A primary antibody against p53 (clone MX008, Fuzhou Maixin Biotechnology Development Co., Ltd.) was used for IHC assay. The detailed protocol for p53 IHC has been described in our previous report (Xu et al., 2023). The p53 expression status was examined microscopically by two independent researchers (Yanjuan Xu and Xueyu Zhuang).

**FIGURE 2:**
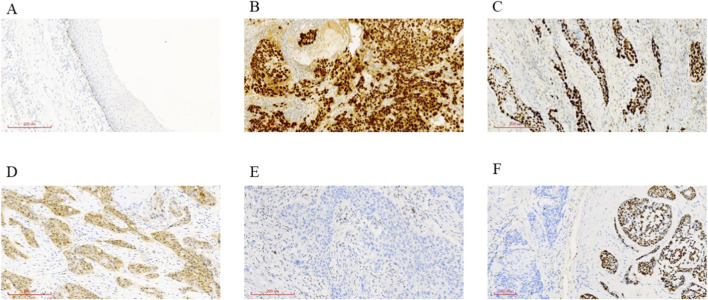
Expression patterns of p53 protein in ESCC. **(A)** Wild-type p53 expression. **(B)** Type I mutant p53 (diffuse internuclear phenotype). **(C)** Type II mutant p53 (basal-cell phenotype). **(D)** Type III mutant p53 (cytoplasmic-positive phenotype). **(E)** Type IV mutant p53 (completely negative phenotype). **(F)** Type V mutant p53 (mixed phenotype).

**FIGURE 3:**
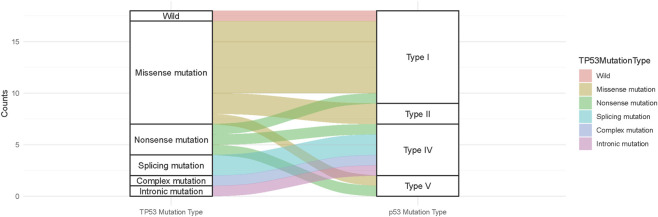
Association between TP53 mutations and mutant p53 expression (Sankey diagram).

According to the protein expression characteristics, the p53 phenotype was classified into wild type (p53^Wild^) and mutant type (p53^mut^) (Xu et al., 2023). p53^Wild^ was defined as 1%–50% of cells in the basal and parabasal layers showing varying intensities of dispersed nuclear positivity, as shown in Figure 2A p53^mut^ can be further classified into four subtypes: (1) Type I (diffuse internuclear mutant phenotype): >50% of nuclei showing diffusely strong positivity, as shown in Figure 2B; (2) Type II (basal-cell mutant phenotype): >50% of basal-layer cells showing strong positivity, as shown in Figure 2C; (3) Type III (cytoplasmic mutant phenotype): cytoplasmic p53 staining with or without nuclear staining, as shown in Figure 2D. (4) Type IV (complete absence of expression): complete lack of detectable p53 expression in the assessed area, as shown in Figure 2E. (5) Type V (mixed mutant phenotype): concurrent features of Types I and IV, as shown in Figure 2F.

### 2.5 Prognostic outcome of TP53 gene in ESCC

The transcriptome RNA-Seq raw counts of ESCC patients were downloaded from TCGA-ESCA. The downloaded samples were divided into TP53 mutation-type and wild-type via “TCGAmutations” and “maftools” R package. Prognostic value of TP53 was analyzed using the Cox proportional risk model via “survminer” and “survival” R package.

### 2.6 Statistical analysis

Statistical analyses were performed using R software (version 4.3.1). Count data, such as gene mutations and mutation sites, are expressed as cases (percentages). The Chi-square test and Kappa statistic were used for analyses (Kappa≥0.7, high consistency; 0.4≤Kappa<0.7, moderate consistency; Kappa<0.4, poor consistency). Statistical significance was set at p < 0.05.

## 3 Results

### 3.1 General pathological features of the patients

The detailed pathological features of the WES, WGS, and IHC cohorts are shown in Table 1.

**TABLE 1:** Detailed pathological features of the WES + WGS cohort and the IHC cohort.

Variables	Variable levels	Total cohort (n = 78)	IHC cohort (n = 60)	WES WGS cohort (n = 18)	t value/F value	P value
Age	Age	65.19 ± 9.31	64.82 ± 9.17	66.44 ± 9.93	0.648	0.519
Gender	Male	52 (66.67)	41 (68.33)	11 (61.11)	0.325	0.569
	Female	26 (33.33)	19 (31.67)	7 (38.89)	​	​
Age group	≤60 years	28 (35.90)	21 (35.00)	7 (38.89)	0.091	0.763
	>60 years	50 (64.10)	39 (65.00)	11 (61.11)	​	​
TNM stage	Stage II	34 (43.59)	27 (45.00)	7 (38.89)	14.495	0.002
	Stage III	28 (35.90)	20 (33.33)	8 (44.44)	​	​
	Stage IVA	3 (3.85)	0 (0.00)	3 (16.67)	​	​
	Stage I	13 (16.67)	13 (21.67)	0 (0.00)	​	​
Tumor gross type	Superficial type	7 (8.97)	7 (11.67)	0 (0.00)	9.263	0.055
	Ulcerative type	25 (32.05)	20 (33.33)	5 (27.78)	​	​
	Medullary type	38 (48.72)	29 (48.33)	9 (50.00)	​	​
	Protruded type	6 (7.69)	4 (6.67)	2 (11.11)	​	​
	Sclerotic type	2 (2.56)	0 (0.00)	2 (11.11)	​	​
Tumor location	Middle thoracic	24 (30.77)	17 (28.33)	7 (38.89)	7.036	0.134
	Lower thoracic	36 (46.15)	25 (41.67)	11 (61.11)	​	​
	Cervical	2 (2.56)	2 (3.33)	0 (0.00)	​	​
	Upper thoracic	1 (1.28)	1 (1.67)	0 (0.00)	​	​
	Esophagogastric junction	15 (19.23)	15 (25.00)	0 (0.00)	​	​
p53 mutation type	Type I	47 (60.26)	38 (63.33)	9 (50.00)	4.282	0.369
	Type II	4 (5.13)	2 (3.33)	2 (11.11)	​	​
	Type III	2 (2.56)	2 (3.33)	0 (0.00)	​	​
	Type IV	21 (26.92)	16 (26.67)	5 (27.78)	​	​
	Type V	4 (5.13)	2 (3.33)	2 (11.11)	​	​

TNM, tumor node metastasis classification.

### 3.2 Detection results of TP53 gene mutations in ESCC tissues

WES showed that TP53 mutations were present in 13/18 (72.22%) patients. Further WGS analysis was performed on the remaining five WES-negative samples, and TP53 mutations were detected in four of them. Overall, TP53 mutations were identified in 17/18 (94.44%) cases. The three most common mutation types were missense mutation (55.56%,10/18), nonsense mutation (16.67%,3/18) and splicing mutation (11.11%, 2/18). The association between TP53 mutation and p53 mutant expression is shown in Figure 3. Summary of TP53 gene mutation information was shown in Supplementary Table S1.

In the WES + WGS cohort, Type III p53 mutation were not detected. The Sankey diagram indicates that missense TP53 mutations were more likely to result in Type I, Type II and Type V mutations in p53. Nonsense TP53 mutations were more likely to result in Type I, Type IV and Type V expression patterns. Other TP53 mutation types were more likely to result in Type IV p53 mutant expression. No Type IV p53 mutations were found in patients with TP53 missense mutations.

### 3.3 Detection results of p53 protein mutations in ESCC tissues

In the WES + WGS cohort, p53 mutant expression was present in all 18/18 (100.00%) patients with ESCC. In the IHC cohort, p53 mutant expression was also present in all 60/60 (100.00%) patients with ESCC. The distribution of p53 mutant expression in both cohorts is shown in Table 1. In both cohorts, Type I was the most common mutant expression pattern, followed by Type IV. Type III mutant expression was not observed in the WES + WGS cohort. The Kappa value between the two pathologists assessing p53 mutant expression was 0.954 (0.911–1.000).

No p53 mutant expression was detected in adjacent tissues from any of the 78 ESCC cases. Therefore, the sensitivity and specificity of p53 mutant expression for diagnosing ESCC were1.00 (0.95–1.00) and 1.00 (0.95–1.00), respectively.

### 3.4 Analysis of clinical characteristics of different p53 mutant expression patterns

We compared the clinical characteristics across the five p53 mutant expression subtypes. There were no significant differences among the subtypes in age, sex, tumor type, gross tumor type, or primary tumor location. The results are presented in Table 2.

**TABLE 2:** Analysis of clinical characteristics across different p53 mutant expression patterns.

Variables	Variable levels	Total	Type I	Type II	Type III	Type IV	Type V	t value/F value	P value
Gender	Male	52 (66.67)	33 (70.21)	2 (50.00)	1 (50.00)	13 (61.90)	3 (75.00)	1.355	0.852
	Female	26 (33.33)	14 (29.79)	2 (50.00)	1 (50.00)	8 (38.10)	1 (25.00)	​	​
AgeGroup	≤60 years	28 (35.90)	17 (36.17)	3 (75.00)	0 (0.00)	6 (28.57)	2 (50.00)	4.615	0.329
	>60 years	50 (64.10)	30 (63.83)	1 (25.00)	2 (100.00)	15 (71.43)	2 (50.00)	​	​
Histological type	ESCC	78 (100.00)	47 (100.00)	4 (100.00)	2 (100.00)	21 (100.00)	4 (100.00)	​	​
TNM	Stage II	34 (43.59)	17 (36.17)	4 (100.00)	2 (100.00)	10 (47.62)	1 (25.00)	9.914	0.623
	Stage III	28 (35.90)	19 (40.43)	0 (0.00)	0 (0.00)	7 (33.33)	2 (50.00)	​	​
	Stage IVA	3 (3.85)	2 (4.26)	0 (0.00)	0 (0.00)	1 (4.76)	0 (0.00)	​	​
	Stage I	13 (16.67)	9 (19.15)	0 (0.00)	0 (0.00)	3 (14.29)	1 (25.00)	​	​
Tumor gross type	Superficial type	7 (8.97)	5 (10.64)	0 (0.00)	0 (0.00)	2 (9.52)	0 (0.00)	14.319	0.575
	Ulcerative type	25 (32.05)	17 (36.17)	0 (0.00)	2 (100.00)	5 (23.81)	1 (25.00)	​	​
	Medullary type	38 (48.72)	18 (38.30)	4 (100.00)	0 (0.00)	13 (61.90)	3 (75.00)	​	​
	Protruded type	6 (7.69)	5 (10.64)	0 (0.00)	0 (0.00)	1 (4.76)	0 (0.00)	​	​
	Sclerotic type	2 (2.56)	2 (4.26)	0 (0.00)	0 (0.00)	0 (0.00)	0 (0.00)	​	​
Tumor location	Middle thoracic	24 (30.77)	12 (25.53)	2 (50.00)	0 (0.00)	9 (42.86)	1 (25.00)	19.195	0.259
	Lower thoracic	36 (46.15)	26 (55.32)	1 (25.00)	0 (0.00)	6 (28.57)	3 (75.00)	​	​
	Cervical	2 (2.56)	2 (4.26)	0 (0.00)	0 (0.00)	0 (0.00)	0 (0.00)	​	​
	Upper thoracic	1 (1.28)	0 (0.00)	0 (0.00)	0 (0.00)	1 (4.76)	0 (0.00)	​	​
	Esophagogastric junction	15 (19.23)	7 (14.89)	1 (25.00)	2 (100.00)	5 (23.81)	0 (0.00)	​	​
Group	IHC only	60 (76.92)	38 (80.85)	2 (50.00)	2 (100.00)	16 (76.19)	2 (50.00)	4.282	0.369
	WES + WGS + IHC	18 (23.08)	9 (19.15)	2 (50.00)	0 (0.00)	5 (23.81)	2 (50.00)	​	​
Age	Age	65.19 ± 9.31	64.62 ± 9.06	59.00 ± 6.53	73.00 ± 7.07	66.90 ± 10.33	65.25 ± 8.81	1.017	0.404

TNM, tumor node metastasis classification.

### 3.5 Prognostic outcome of TP53 gene in ESCC

**FIGURE 4:**
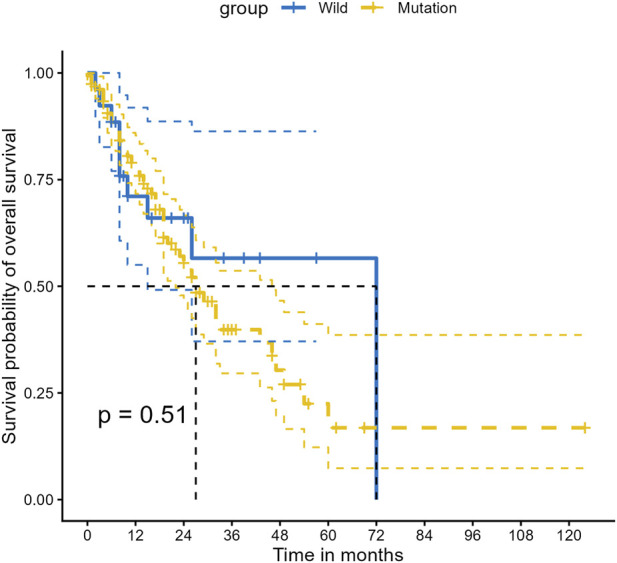
Kaplan–Meier survival analysis of TP53 mutations on survival in human ESCC.

Kaplan-Meier survival analysis showed that overall survival proportion at 3-years and 5-years were 56.58% (37.09%–86.31%) vs. 39.84% (29.59%–53.63%) and 56.58% (37.09%–86.31%) vs. 16.85% (7.36%–38.59%), respectively, for TP53 wild group and TP53 mutation group, without statistical significance [hazard ratio = 1.25 (0.64–2.45), *P* = 0.517]. The results are presented in Figure 4.

## 4 Discussion

This study yielded several important discoveries. First, we identified TP53 gene mutation as a key pathological event in ESCC, with an extremely high mutation rate. Second, TP53 gene mutations and mutant p53 protein expression demonstrated relatively high pathological concordance. Third, and most notably, we identified for the first time five easily recognizable p53 mutant expression patterns in ESCC that can produce consistent diagnostic interpretations among different pathologists. Finally, mutant p53 protein expression represents an important pathological feature of ESCC and can serve as a useful reference in its diagnosis.

With advancements in modern molecular technologies, the detection of gene expression and mutation status in patient with cancer has become feasible in clinical practice. These genetic findings enable the rapid development of molecular targeted therapies and support the formulation of personalized antitumor treatment strategies. Therefore, identifying clinically relevant cancer-related genetic targets is essential for the development of targeted therapeutic agents.

The TP53 gene, first reported by Lane et al., in 1979, encodes a protein with a molecular weight of approximately 53 kDa, known as p53 (Lane et al., 1979). Based on its functional status, TP53 can be classified into wild-type and mutant forms. Recent studies have shown that cancer development is associated with abnormalities in multiple tumor-related genes, among which TP53 mutations are particularly common. TP53 gene mutations have been frequently identified in papillary thyroid carcinoma, prostate cancer, lung cancer, breast cancer, pancreatic cancer, liver cancer and malignant melanoma (Mogi and Kuwano, 2011; Malcikova et al., 2014; Xu et al., 2022; Qayoom et al., 2024). Wild-type TP53 is one of the most important tumor suppressor genes and is often referred to as “the guardian of the genome” (Giacomelli et al., 2018; Voskarides and Giannopoulou, 2023). It plays crucial roles in regulating the cell cycle, inhibiting DNA synthesis, repairing DNA damage, and regulating apoptosis (Voskarides and Giannopoulou, 2023; Ray Das et al., 2024). In contrast, mutant TP53 functions as a proto-oncogene (Mogi and Kuwano, 2011; Malcikova et al., 2014). The p53 protein encoded by TP53 acts as a transcription factor and can inhibit other tumor suppressor genes, thereby promoting alterations in oncogenic signaling pathways and increasing the risk of tumorigenesis (Xu et al., 2022; Voskarides and Giannopoulou, 2023; Ray Das et al., 2024). The growth advantage of transformed cells and tumors arises from the inactivation or loss of the TP53 caused by gene mutations. Thus, detecting alterations in TP53 is biologically meaningful for understanding the initiation and progression of ESCC.

Previously, the primary method for detecting TP53 mutations was WES(27). Under this approach, the mutation rate of TP53 in ESCC was generally between 60% and 75% (Zhong et al., 2023; Abedi-Ardekani et al., 2011). Recently, several large-scale genomic sequencing studies have shown that ESCC harbors a TP53 mutation rate of up to 90% (Qin et al., 2016; Soussi and Béroud, 2001; Lin et al., 2014). In our study, WES identified TP53 mutations in 13/18 (72.22%) cases, which aligns with previous reports (Zhong et al., 2023; Abedi-Ardekani et al., 2011). With the aid of next-generation sequencing, we further detected rare TP53 mutations through WGS, including intron variants. This approach increased the mutation detection rate from 13/18 (72.22%) to 17/18 (94.44%), indicating that combined WGS and WES testing may enhance the detection of TP53 mutations. Although additional mutation types were identified, the clinical significance of these rare variants remain unclear.

After mutations occur in the TP53 gene, alterations in the spatial conformation of the p53 protein affect its transcriptional activation and phosphorylation functions. Consequently, not only is the tumor-suppressive effect of wild-type TP53 lost, but the mutation itself may also confer oncogenic properties, disrupting the transcriptional regulation of multiple oncogenes and promoting tumor development (Kamada et al., 2011). Tumors harboring TP53 mutations often exhibit higher malignancy, and elevated p53 expression is associated with poorer survival in ESCC(33, 34). In normal tissues, wild-type p53 degrades rapidly and has a short half-life; thus, IHC staining shows positivity in only a subset of cells (Kamada et al., 2011). Under physiological conditions, p53 IHC typically presents as scattered nuclear positivity of varying intensity. When the mutant p53 protein undergoes conformational changes, its half-life is significantly prolonged, resulting in strong IHC staining. The current criterion for strong p53 positivity is that more than 60% of tumor cells exhibit diffuse, intense nuclear staining under high-power microscopy, at which point the protein expression pattern is considered indicative of a mutant type (Chen et al., 2022b; Mahat et al., 2025). TP53 mutations generally correlate well with positive p53 IHC staining because most TP53 mutations are missense variants. In contrast, nonsense mutations produce truncated proteins that lack antigenic epitopes necessary for antibody binding, leading to completely negative IHC staining, as shown in Figure 2E. In our study, the three most common mutation types were missense (55.56%, 10/18), nonsense (16.67%, 3/18) and splicing mutations (11.11%, 2/18).

TP53 gene mutations are common in ESCC, and mutant expression of the p53 protein has been shown to have a high degree of concordance with TP53 mutations (Zhong et al., 2023; Qin et al., 2016). Previous studies have reported that the expression of mutant p53 protein in ESCC tissues is significantly higher than that in normal tissues, resulting in much stronger IHC staining intensity in cancer tissues (Guan et al., 2023). Although mutant p53 expression has been widely described; specific subtypes have not yet been clearly defined (Zhang et al., 2020; Hu et al., 2021). In the present study, we identified five mutant p53 protein subtypes with distinct expression patterns. These subtypes exhibit unique microscopic characteristics that can be readily recognized by pathologists. Consistent with previous findings, the interobserver agreement for p53 subtype classification was high, with a kappa value of 0.954 (0.911–1.000). Our results showed that missense mutations in TP53 were more likely to cause Type I, Type II, and Type V p53 expression patterns. Nonsense mutations were more likely to lead to Type I, Type IV, and Type V expressions patterns, while other TP53 mutations were more likely to produce Type IV expression patterns. Although the clinical significance of these five subtypes has not yet been established, our findings provide a classification framework for pathologists to support further investigations of mutant p53 expression. In a previous report, p53 expression was scored as overexpression (OE), complete absence (CA), cytoplasmic (CY) or wild type (WT) in ovarian carcinoma (Köbel et al., 2016) based on IHC staining. It was found that p53 IHC can reliably predict TP53 mutations independently identified by next-generation sequencing (Köbel et al., 2016). Similar results showed that optimized p53 IHC performs well as a surrogate test for TP53 mutation in endometrial carcinoma biopsies, demonstrates excellent inter-laboratory reproducibility, and has high clinical utility for molecular classification algorithms in endometrial carcinoma (Singh et al., 2020). In addition, it was found that p53 IHC pattern reasonably predicted TP53 mutational status in solid cancer cases (Sung et al., 2022).

The diagnostic value of p53 in ESCC has garnered increasing attention (Shimada, 2018; Chen et al., 2022b). TP53 mutations are the most common genetic alterations associated with human cancer, and TP53 missense mutations—which frequently occur early in ESCC—are present in over 50% of cases (Zhong et al., 2023). Therefore, TP53 mutation is an important molecular feature of ESCC and has potential diagnostic value. Most TP53 mutations result from amino acid substitutions within the core region of the p53 protein, generating various forms collectively known as “mutant p53.” In addition to acquiring oncogenic properties through gain-of-function pathways, these mutants also lost typical tumor-suppressive functions to varying degrees. As a result, mutant p53 expression can be readily detected in clinical practice using IHC staining. In this study, we found that mutant p53 expression was present in 18/18 (100.00%) cases in the WES + WGS cohort and in 60/60 (100.00%) cases in the IHC cohort. In contrast, none of the adjacent tissues from the 78 ESCC cases showed mutant p53 expression. These findings indicate that mutant p53 expression is a defining feature of ESCC tissues and demonstrates extremely high sensitivity and sensitivity for distinguishing cancerous from noncancerous tissues.

The gain-of-function effect of stabilized mutant p53 contributes to tumor-specific dependency and resistance to therapy. P53 remains a prospective target for cancer treatment because of its tumor-suppressive functions and the wide range of alterations it undergoes in tumors. Phenotypic abnormalities in breast cancer, notably in poorly differentiated basal-like tumors, are frequently associated with high-grade disease. By integrating data from cell and animal models with clinical outcomes in breast cancer, this study investigated the molecular mechanisms through which gene alterations lead to the pathogenic consequences of mutant p53 tumorigenic activity. This study also examines current and emerging therapeutic strategies targeting p53 mutations, considering both the shared and distinct regulatory mechanisms of mutant and wild-type p53.

The relationship between mutant p53 expression levels and the prognosis of ESCC has also gained attention (Jin et al., 2023; Zhao et al., 2017). The mutation rate of p53 in ESCC tissues is significantly higher than that in normal tissues. Mutant p53 loses its ability to inhibit tumor growth, thereby promoting tumor initiation and progression and causing cancer cells to exhibit increased invasiveness and accelerated disease advancement. Consequently, patients with ESCC with a high p53 mutation rate experience significantly increased mortality and reduced long-term survival (Jin et al., 2023; Zhao et al., 2017). Emerging evidence indicates that mutant p53 is strongly associated with advanced malignancy and poor prognosis, making it an attractive target for the development of novel cancer therapies (Parrales and Iwakuma, 2015; Capaci et al., 2020). Previous studies have shown that mutant p53 may serve as an important indicator for evaluating the prognosis of malignant tumors (Butera and Amelio, 2025). Imamhasan et al. reported reduced survival in patients with ESCC with higher frequencies of p53 mutations compared with those with common ESCC (Imamhasan et al., 2012), and similar findings have been demonstrated in another study (Yao et al., 2014).

Our study has several limitations. First, due to limited research funding, we completed WES in only 18 patients with ESCC and WGS in five patients with ESCC. The small sample size may have prevented the detection of all mutation types. Second, our study only distinguished mutant p53 types solely based on IHC staining characteristics. Whether this classification has clinical value requires further investigation, such as determining whether gene expression profiles differ among subtypes using transcriptome sequencing. Third, the exclusion of patients who received preoperative therapy may have biased the cohort toward early-stage disease, thereby limiting the generalizability of the findings. The TNM stage distribution was uneven (Table 2), which may serve as a potential confounder. Finally, our study did not thoroughly examine the prognostic impact of mutant p53 or compare prognostic differences among subgroups. Therefore, future work should include systematic collection of survival data to enable prognostic analyses.

## 5 Conclusion

Mutant-expressing p53 can serve as an alternative marker for TP53 mutation screening using WES or WGS. Mutant p53 expression shows relatively high sensitivity and specificity in distinguishing ESCC and can aid in differentiating benign from malignant esophageal lesions. Five different mutant p53 protein subtypes were identified in this study; however, their clinical significance requires further investigation.

## Data Availability

The original contributions presented in the study are publicly available. This data can be found at the NCBI repository with the accession number PRJNA1425652.
